# Impact of Nanocomposite Combustion Aerosols on A549 Cells and a 3D Airway Model

**DOI:** 10.3390/nano11071685

**Published:** 2021-06-27

**Authors:** Matthias Hufnagel, Nadine May, Johanna Wall, Nadja Wingert, Manuel Garcia-Käufer, Ali Arif, Christof Hübner, Markus Berger, Sonja Mülhopt, Werner Baumann, Frederik Weis, Tobias Krebs, Wolfgang Becker, Richard Gminski, Dieter Stapf, Andrea Hartwig

**Affiliations:** 1 Department of Food Chemistry and Toxicology, Institute of Applied Biosciences, Karlsruhe Institute of Technology, 76131 Karlsruhe, Germany; Matthias.Hufnagel@gmail.com (M.H.); Johanna.Wall@kit.edu (J.W.); 2 Institute for Technical Chemistry, Karlsruhe Institute of Technology, 76344 Eggenstein-Leopoldshafen, Germany; nadine.may@kit.edu (N.M.); sonja.muelhopt@kit.edu (S.M.); werner.baumann@kit.edu (W.B.); 3 Institute for Infection Prevention and Hospital Epidemiology, Medical Center—University of Freiburg, Faculty of Medicine, University of Freiburg, 79110 Freiburg, Germany; nadja.wingert@uniklinik-freiburg.de (N.W.); manuel.garcia-kaeufer@uniklinik-freiburg.de (M.G.-K.); ali.arif@uniklinik-freiburg.de (A.A.); richard.gminski@uniklinik-freiburg.de (R.G.); 4 Fraunhofer Institute of Chemical Technology, 76327 Pfinztal, Germany; christof.huebner@ict.fraunhofer.de (C.H.); wolfgang.becker@ict.fraunhofer.de (W.B.); 5 Vitrocell® Systems GmbH, 79183 Waldkirch, Germany; m.berger@vitrocell.com (M.B.); t.krebs@vitrocell.com (T.K.); 6 Palas GmbH, 76229 Karlsruhe, Germany; frederik.weis@palas.de

**Keywords:** nanocomposite, nano-enabled polymer (NEP), nanoparticles, air-liquid interface (ALI), nanotoxicology, incineration

## Abstract

The use of nanomaterials incorporated into plastic products is increasing steadily. By using nano-scaled filling materials, thermoplastics, such as polyethylene (PE), take advantage of the unique properties of nanomaterials (NM). The life cycle of these so-called nanocomposites (NC) usually ends with energetic recovery. However, the toxicity of these aerosols, which may consist of released NM as well as combustion-generated volatile compounds, is not fully understood. Within this study, model nanocomposites consisting of a PE matrix and nano-scaled filling material (TiO_2_, CuO, carbon nano tubes (CNT)) were produced and subsequently incinerated using a lab-scale model burner. The combustion-generated aerosols were characterized with regard to particle release as well as compound composition. Subsequently, A549 cells and a reconstituted 3D lung cell culture model (MucilAir™, Epithelix) were exposed for 4 h to the respective aerosols. This approach enabled the parallel application of a complete aerosol, an aerosol under conditions of enhanced particle deposition using high voltage, and a filtered aerosol resulting in the sole gaseous phase. After 20 h post-incubation, cytotoxicity, inflammatory response (IL-8), transcriptional toxicity profiling, and genotoxicity were determined. Only the exposure toward combustion aerosols originated from PE-based materials induced cytotoxicity, genotoxicity, and transcriptional alterations in both cell models. In contrast, an inflammatory response in A549 cells was more evident after exposure toward aerosols of nano-scaled filler combustion, whereas the thermal decomposition of PE-based materials revealed an impaired IL-8 secretion. MucilAir™ tissue showed a pronounced inflammatory response after exposure to either combustion aerosols, except for nanocomposite combustion. In conclusion, this study supports the present knowledge on the release of nanomaterials after incineration of nano-enabled thermoplastics. Since in the case of PE-based combustion aerosols no major differences were evident between exposure to the complete aerosol and to the gaseous phase, adverse cellular effects could be deduced to the volatile organic compounds that are generated during incomplete combustion of NC.

## 1. Introduction

The number of plastic products containing nanomaterials increases continuously. The annual worldwide production of polymers is currently around 360 million tons, with 60 million tons produced in Europe and 20 million tons in Germany, including products such as adhesives and foams. In the field of thermoplastics, Germany’s annual production volume is about 12 Mt and every year about 6 Mt of plastic waste are generated in Germany, most of which is recovered energetically. The chemical industry in Germany is a leading manufacturer of plastic precursors in Europe and bears a significant share of the responsibility for a change from a linear economy with large quantities of waste to a closed-loop economy with high recycling volumes. Estimates assume strong growth in plastics production, which is expected to be around 500 million t/a in 2025. For 2050, per capita consumption in industrialized countries is predicted to be around 150 kg/a, which would correspond to a massive increase in production.

Thermoplastics generally have a high proportion of additives, some in nanoparticulate form. During recycling, but especially during energy recovery, these nanoparticulate additives can be released [1]. Due to their small size and the resulting large specific surface area, nanomaterials have a number of specific mechanical and physical properties. By using them as fillers, these properties can be specifically transferred to polymers. Compared to conventional composites, the resulting nanocomposites have a greatly improved property profile that cannot be achieved with conventional fillers or only with significantly higher filler contents. Although the term nanocomposite (NC) suggests a certain novelty, nano-scaled fillers have been used as classic additives in polymer processing for many years. The best-known example is certainly carbon black, which is widely used to increase UV stability and electrical conductivity [2]. Exact figures on the extent to which nano-scaled fillers are already used today are hardly available, which can certainly also be attributed to a lack of mandatory declaration for nanomaterials [3]. However, it is reported that nanoparticulate substances are already widely used in plastics technology. Every year, several million tons of titanium dioxide (TiO_2_) are processed worldwide, including a certain percentage of nano-scaled material [4] for the production of plastics, but also lacquers, paints, food, cosmetic products, and pharmaceuticals. In Germany, around 0.142 million tons of TiO_2_ were processed into 14.4 million tons of plastic products in 2017. Based on all plastic applications, the average TiO_2_ content is 1% and over 60% of the total TiO_2_ quantity is processed in plastic applications in the construction and packaging industry.

The growing amount of plastic waste is leading to worldwide environmental problems, such as marine litter and microplastics, resulting in a global challenge. Industrialized nations, which are the main producers of these products, are where plastics are particularly in demand. As demanded in several reports, immediate action is required for prevention, safe handling, recycling, and orderly disposal. In industrialized regions there is usually a functioning waste management system so that plastic waste can be collected and either be recycled or be recovered energetically via incineration. In recent studies, the thermal utilization of nanocomposites is regarded as the relevant end-of-life scenario [5,6,7,8]. Even though studies on the release of particulate matter during this end-of-life scenario have been published for CNT- and nanoparticle-based materials, e.g., [5,6,9,10,11], research on the toxicological impact of byproducts is still scarce, especially with regard to the volatile gaseous phase generated during this process [1,7,12,13]. In modern incineration plants with a state-of-the-art flue gas cleaning system, there is no danger of airborne particles being released [14]. In many countries of the world, however, plastic waste is largely deposited into the environment. In such places, uncontrolled incineration for waste disposal, resulting in an exposure of humans and the environment toward the combustion aerosols, is an everyday problem.

Most studies published so far investigating the toxicological effects of aerosols in vitro are based on the submerged exposure of test systems to collected particulate matter suspended in the cell culture medium. This approach was used previously to investigate the toxicity of incineration byproducts [1,7,12,13]. However, this methodology neglects the incineration-generated gaseous phase, which has to be considered to reveal a comprehensive toxicological impact [10,11,12]. Moreover, suspending collected material in an aqueous cell culture medium may change the properties of the investigated particles by medium-particle interactions, resulting in a protein corona [15] and partial solubility and does not reflect the impact of the human lung, as pointed out by the OECD [16]. Thus, experts and the OECD stated that exposure at the air–liquid interface (ALI) between cell cultures and aerosols is the method of choice for future investigations [17,18]. ALI avoids many disadvantages but requires a well-established and characterized system to guarantee reproducible conditions. Therefore, KIT and VITROCELL Systems developed a fully automated ALI exposure station that offers a complete measurement system for the parallel exposure of up to 24 human lung cell culture samples toward gases, nanoparticles, and complex mixtures such as combustion aerosols [19]. The particle mass per area deposited via diffusional as well as via electrostatic mechanism for dose enhancement can be monitored online using a quartz crystal microbalance [20,21]. Additionally, a new tool to reproducibly expose sample grids for transmission electron microscopy (TEM) to obtain dose information with respect to the spatial distribution and the agglomeration state of the deposited particles was developed and applied within this study [22]. The ALI exposure station has already been used to investigate the toxicological impact of environmental atmospheres and technical emission sources like ship diesel exhaust [23,24], biomass combustion emissions [25], and suspended nanomaterials as metal oxides and silica [15,26,27].

The aim of the present study was to develop a methodology to investigate the behavior and toxicological impact of nanocomposite released byproducts during thermal decomposition. Therefore, respective materials were produced using TiO_2_, CNT, and CuO (all 10 wt%) as nano-scaled fillers and polyethylene (PE) as a matrix. TiO_2_ and CNT were chosen due to their industrial use in nanocomposites, whereas CuO was chosen as a reference material because of its well-known toxicological impact [28,29]. Therefore, it was hypothesized that particulate matter generated from PE + CuO nanocomposite incineration might induce a more toxic reaction than the TiO_2_-containing nanocomposite. Subsequently, all materials were incinerated using a lab-scale burner and the resulting aerosol was characterized with regard to size, concentration, and morphology. The incineration byproducts were further analyzed for their toxicological potential using the described ALI exposure station and an epithelial cell monolayer (A549) as well as a more sophisticated and realistic reconstituted lung cell culture model (MucilAir™). The following exposure scenarios were considered: (a) exposure under normal conditions, (b) exposure under conditions of enhanced particle deposition, and (c) exposure toward a filtered aerosol and therefore gaseous phase only. Toxicological profiles of all exposure scenarios were investigated with regard to cyto- and genotoxicity, inflammatory response, and transcriptional toxicity profiling using high-throughput RT-qPCR [30].

## 2. Materials and Methods

### 2.1. Combustion of Nanomaterials and Nanocomposites

#### 2.1.1. Nanomaterials

For thermal decomposition of nano-scaled filler material, TiO_2_ (Aeroxide^®^ P25, Evonik, Essen, Germany), multi-wall CNTs (NC7000™, Nanocyl^®^, Sambreville, Belgium), and CuO (product number 544868, Sigma-Aldrich^®^, Munich, Germany) were used, each of which was applied as a suspension with deionized water. In each case, 4 g of the nanomaterial were dispersed in 1 L of deionized water and treated with ultrasound for one hour. In the case of CNTs a stable suspension with water was not achievable, therefore a stabilizer, 10 g/L gum arabic, was added to the suspension.

#### 2.1.2. Nanocomposites

For the production of the nanocomposites, the nanomaterials were fed as delivered in the compounding process. In this investigation, TiO_2_ compounds were produced in a Leistritz 27 HP extruder (Leistritz Extrusionstechnik GmbH, Nuremberg, Germany) with a 27 mm screw diameter and an L/D of 52. CNT and CuO compounds were produced in a Leistritz ZSE 18 MAXX extruder (Leistritz Extrusionstechnik GmbH, Nuremberg, Germany) with an 18 mm screw diameter and an L/D of 60. Both machines were equipped with a special encapsulated dosing technique for processing of nanoparticles, avoiding dust via a special sealing technique for refilling the gravimetric feeders. The produced compounds were characterized in view of mechanical properties on injection molded samples according to DIN EN ISO 527 and the filler content was checked via TGA.

#### 2.1.3. Burner and Aerosol Conditioning

For this study, a laboratory Bunsen-type burner (constructed by KIT, Karlsruhe, Germany) was used to represent the thermal decomposition of end-of-life nanocomposites. Either nanocomposite powders or nanoparticle suspensions were added to the feed gas stream of the burner. A rotating brush generator (RBG1000, Palas, Karlsruhe, Germany) was used for the dosage of the nanocomposite powders and an atomizer (ATM220, Topas, Dresden, Germany) was used for the experiments with pure nanoparticles. For a smooth operation of the rotating brush generator, the nanocomposites were sieved with a 315 µm sieve (Retsch, Haan, Germany) and the powder fraction with sizes smaller than 315 µm was used. The material reservoir was filled with approximately 4 g of NC powder and the feed rate was adjusted to 1 g/h. For the experiments with pure nanoparticles, suspensions with 4 g/L solid material and deionized water were prepared. The air volume flow for atomization was set at 1 l_N_/min, which led to a dosing of about 4 g/h suspension.

**Figure 1:**
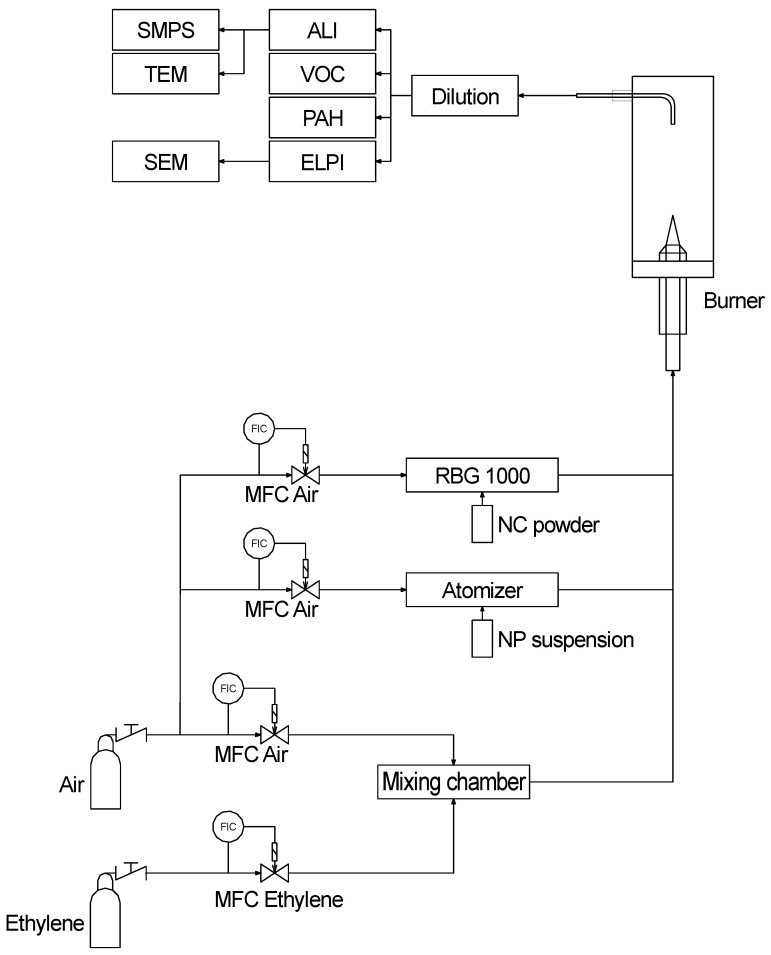
Setup for the thermal degradation of nanocomposite powders and nanoparticle suspensions with subsequent physicochemical and toxicological characterization.

The burner was operated with a premixed ethylene/air burning gas mixture with slightly over-stoichiometric conditions (λ_gas_ = 1.07). The dosage of nanocomposites reduced the air number by about 2%. The total airflow was set to 9.30 l_N_/min and the ethylene flow to 0.61 l_N_/min controlled via mass flow controllers (EL-Flow, Bronkhorst, Ruurlo, The Netherlands). At 430 mm above the burner, a sampling probe was installed, followed by a dilution stage (VKL10E, Palas, Karlsruhe, Germany). The dilution stage diluted the aerosol 10-fold on the one hand to decrease the temperature after the combustion and on the other hand to increase the available volume flow. Downstream of the dilution stage, the different systems for the aerosol characterization as well as human lung cell exposure were installed (Figure 1).

#### 2.1.4. Characterization of Combustion Aerosols

For the aerosol measurement, an electrical low-pressure impactor (ELPI, Dekati, Kangasala, Finland) was installed downstream of the dilution stage. The ELPI measures charged particles in the size range of 6 nm to 10 µm and the deposited particles can be used for subsequent analyses. The ELPI is a low-pressure cascade impactor to which one electrometer per impactor stage is connected, which records the current of the impacted charged particles [31]. The particles are charged with a corona before entering the cascade impactor, then classified according to their inertia and their number is determined by the measured current per stage. The individual impactor stages on which the particles are deposited are, for example, occupied with aluminum foils, which can be used for imaging or chemical analysis after the measurement. The stage at which a particle impacts depends on its aerodynamic diameter, which is affected by particle size, shape, and density. The measurement results in a number size distribution.

The ELPI was equipped with aluminum foil substrates at each stage for the size-classified collection of particles that can be examined via scanning electron microscope (Zeiss Supra 55VP SEM with a field emission gun operating at 3 kV and 10 kV accelerating voltage and an aperture of 30 µm) (Carl Zeiss Microscopy Deutschland GmbH, Oberkochen, Germany). Every second the complete particle size distribution was logged in dependence of the aerodynamic diameter and for further processing the time averaged value can be used. Since the ELPI measurement principle is based on the number concentration, the density of the particles is required to calculate the resulting mass concentration.

The characterization of the combustion gases from combustion of polyethylene (PE) fine granulate was carried out after adsorption of 2 L of the combustion-generated gaseous phase at 37 °C over 20 min on TENAX TA tubes (Merck KGaA, Darmstadt, Germany) using an air collection pump. The subsequent quantitative analysis followed in accordance with DIN ISO 16000-6: 2012-11 using thermal desorption combined with capillary gas chromatography and mass spectrometry. Using the external standard method, individual substances were quantified by including reference substances. Non-quantifiable substance peaks were compared with reference spectra from spectral libraries. Semi-volatile organic compounds (SVOC) were calculated as toluene equivalents (TE) and added up. The results are given as the sum of total volatile organic compounds (TVOC) and of SVOC.

At the reactor of the automated ALI exposure station, a scanning mobility particle sizer (SMPS + C, Grimm, Ainring, Germany) was installed to measure the generated aerosol as it was applied onto cell culture systems. With the SMPS, a number size distribution was measured in dependence of the mobility diameter, which has to be distinguished from the aerodynamic diameter the ELPI is measuring. The particles used for the determination of the particle size by ELPI were collected directly after dilution with dry air and were thus not affected by humidity. In addition, since none of the investigated particles were hygroscopic, no impact of humidity would be expected with respect to TEM images. In one of the exposure chambers of the ALI exposure station, Formvar film-coated copper grids with 200 mesh, type SF162 (Plano GmbH, Wetzlar, Germany), were installed to get an optical evaluation of the deposited particles via transmission electron microscopy (EM 109 (Carl Zeiss Microscopy Deutschland GmbH, Oberkochen, Germany).

The occurrence of polycyclic aromatic hydrocarbons (PAHs), which presumably are condensed on the solid–particulate phase of the aerosol as a result of the combustion process of the (unfilled) PE-matrix, was evaluated exemplarily. Particulate matter (PMx) and condensates were sampled by filtering an aerosol volume of x m³ on a glass fiber plane filter at 37 °C. US EPA priority polycyclic aromatic hydrocarbons (16 PAHs) were quantified in total, placing untreated blank (pure glass fiber filters) and the PM sample in a glass vial containing 10 mL of dichloromethane (DCM) and kept in an ultrasonic bath for 30 min. The extract was then filtered through membrane filters (PTFE 45 µm) and again placed in 10 mL of DCM for ultrasonic extraction. Subsequently, 3 mL acetonitrile was added and the extract was again concentrated to 1 mL in a nitrogen stream. The PAH contents in the extract were determined via high-performance liquid chromatography (HPLC).

#### 2.1.5. Dose Determination

Usually, the dose can be determined via quartz crystal microbalance, but in this study the deposited particle masses were found to be below the detection limits. Kaur and colleagues state the detection limit of QCM to be 50 ng/cm² [32], but the doses in this study were lower. Therefore, the applied particulate dose was determined by using the measured number concentration obtained via an ELPI by calculating the mass concentration with an assumed value for the particle density and shape. The real NP density can differ significantly from the density value of the macroscopic material (bulk). Particles often do not exist individually but as agglomerates. Potential agglomerates are assumed to contain internal voids, which means that the agglomerate takes up a large volume with a comparably small mass, which decreases their density value. For the metal oxide particles alone subjected to thermal treatment, it can be seen from the TEM and SEM (Supplementary Figure S1) images that they are essentially primary particles with an approximately spherical shape, so the respective bulk densities were used for calculation (6.48 g/cm³ for CuO and 4.24 g/cm³ for TiO_2_). In the case of nanocomposites, dose assessment is more complex because the aerosol consists of a mix of different degradation products after combustion. Carbon black is often given with a density of 2 g/cm³, while the unburned plastic has a density of 1 g/cm³. Therefore, a density of 1 g/cm³ and a dynamic shape factor of 1 was assumed for the thermoplastic matrix, nanocomposites, CNTs, and gum arabic (stabilizer).

The total mass of all impactor stages was added and related to the sample stream, resulting in a mass concentration. Using the duration of an experiment (t = 240 min), the area of the transwell membrane, the deposition efficiency, as well as the volume flow over the membrane, the area load could be calculated. The deposition efficiency was known through former studies [26,33] and was found to be approximately 2% by diffusional deposition and 10% by electrostatic deposition.

### 2.2. Cell Culture

A549 cells, alveolar epithelia cells originated from a human adenocarcinoma, were kindly provided by Dr. Roel Schins (Leibniz Research Institute for Environmental Medicine, Düsseldorf, Germany). Cells were cultured as monolayers in RPMI supplemented with 10% fetal bovine serum (FBS), 100 U/mL penicillin, and 100 µg/mL streptomycin and incubated at 37 °C in a humidified atmosphere of 5% CO_2_ (HeraSafe, Thermo Scientific, Langenselbold, Germany). For ALI exposure, 650,000 cells in 1 mL cell culture media were seeded on the apical side of a 24 mm transwell with 1.5 mL cell culture media on the basal side of the transwell. Cells were cultured for 24 h, checked for confluency via microscopy, and washed with phosphate buffered saline (PBS; Carl Roth GmbH, Karlsruhe, Germany). Finally, PBS was removed, providing an ALI on the apical side of the transwell. Cells were subsequently transferred to the respective exposure chamber of the ALI exposure system. Each chamber was filled with 6.5 mL cell culture medium supplemented with 25 mM of HEPES, providing pH stability during exposure. Reconstituted lung tissue was purchased as MucilAir™ from Epithelix (Geneva, Switzerland). The model was cultured in the cell culture medium provided by the supplier. Since experiments with this model were limited, only one nanomaterial and its respective nanocomposite were investigated. The most commonly used nanoparticle for nanocomposites of the three reference materials, TiO_2_ NP, was selected. Before ALI exposure, the tissue culture was washed with PBS on the apical side. The medium was also supplemented with 25 mM of HEPES during ALI exposure.

#### 2.2.1. Air-Liquid Interface Exposure

The native aerosol sample was taken from the process at a volume flow of 1 m³/h and was conducted through a PM10 low-volume impactor. The relative humidity was adjusted to 85% r.H. through water vapor dosing to protect the cell cultures from drying out. The humidified aerosol flowed into the particle reactor where isokinetic sampling probes were installed to conduct the aerosol into the exposure chambers of the VITROCELL modules with a flow rate of 100 mL/min. As a negative control, one module (clean air control—CAC) was supplied with emission-free, humidified air. To increase deposition efficiency, an electrical field was applied between the cell culture surface and the aerosol inlet. To achieve this, in the isolated housing, an electrode was implemented in the bottom of the medium reservoir and supplied with a high voltage of 1000 volts. The cells were exposed for 4 h to the respective aerosol and post-incubated for a further 20 h in an incubator before cytotoxicity, genotoxicity, inflammatory response, and gene expression were determined. Cytotoxicity and inflammatory response were investigated using the basal cell culture medium after 20 h post-incubation. Genotoxicity and gene expression analyses were performed after harvesting the cells.

#### 2.2.2. LDH Release

Cytotoxicity was determined using the Cytotoxicity Detection Kit (LDH) (Roche Diagnostics GmbH Roche Applied Science, Mannheim, Germany), measuring the activity of the cytoplasmic enzyme lactate dehydrogenase (LDH). LDH is a stable cytoplasmic enzyme present in all cells. When cells are undergoing apoptosis, necrosis, and other forms of induced structural cellular damage, LDH is released in the cell culture medium and can be detected colorimetrically. Use of the Cytotoxicity Detection Kit was performed according to the manufacturer’s protocol, analyzing the basal medium collected after 20 h post-incubation. An incubation with 0.1% Triton X-100 for 10 min was used as positive control.

#### 2.2.3. Alkaline Unwinding

DNA strand breaks were quantified after ALI exposure using alkaline unwinding as described previously [29]. Briefly, cells were incubated as described for the respective experiments, trypsinized and resuspended in cold PBS supplemented with 10% FBS. Subsequently, cells were centrifuged at 1,300 rpm at 4 °C for 3 min, washed twice with PBS, and finally suspended in PBS at a cell concentration of 10^6^ cells/mL. Ten microliters of the cell suspension were pipetted into a 15 mL reaction tube and 750 µL of an alkaline solution (pH 12.3; 0.03 M NaOH, 0.02 M Na_2_HPO_4_, 0.9 M NaCl) were added. DNA was unwound for 30 min in the dark followed by neutralization to pH 6.8 adding 0.1 M HCl. After a 15 s sonification, 15 µL of 10% SDS solution were added to the suspension. Single- and double-stranded DNA were separated using hydroxyapatite columns at 60 °C and 0.15 M as well as 0.35 M potassium phosphate buffer for DNA elution, respectively. Finally, 7.5 × 10^−7^ M Hoechst 33258 was given to all samples, followed by detection of fluorescence emissions (excitation 360 nm, emission 455 nm) using an Infinite M200 Pro (Tecan, Männedorf, Switzerland). The amount of DNA strand breaks per base pairs (bp) was calculated as described previously [34].

#### 2.2.4. Gene Expression Analysis

Gene expression analysis was approached using an HT RT-qPCR with Fluidigm Dynamic Arrays on the BioMark™ System (Fluidigm Corperation, San Francisco, USA) as described previously [30]. Data were analyzed using Fluidigm Real-Time PCR Analysis (v4.1.3, Fluidigm Corperation, San Francisco, USA) as well as GenEx (v5, MultID, Goeteborg, Sweden) software. Normalization was performed using five reference genes (*ACTB*, *B2M*, *GAPDH*, *GUSB*, and *HPRT1*). Gene transcription is displayed as a log_2_-fold change compared to a control group exposed to clean air (CAC) by calculating relative quantities corresponding to the ∆∆Cq method [35].

#### 2.2.5. Inflammatory Response

The inflammatory response was determined via the quantitative enzyme-linked immunosorbent assay (ELISA) Human IL-8 ELISA Ready-SET-Go!^®^ (2nd Generation) (Invitrogen, Fisher Scientific, Schwerte, Germany) measuring the secretion of IL-8 (CXCL8), a pro-inflammatory CXC chemokine. IL-8 is expressed by monocytes, macrophages, epithelial cells, and fibroblasts in response to inflammatory stimuli. The Human IL-8 Ready-SET-Go! ELISA Set was performed according to the manufacturer’s protocol. For the positive control, A549 cells were stimulated with 20 ng/mL TNF-a for 20 h and MucilAir™ was stimulated with 0.5 µg/mL TNF-α for 20 h.

### 2.3. Statistical Analysis

If not stated otherwise, all data are displayed as the mean of three independently performed experiments, each of which had been conducted at least in duplicates. Differences on a cellular level between the negative control (treated with clean air) and the aerosol-treated samples were analyzed via one-way ANOVA followed by Dunnet’s post hoc test for multiple comparisons.

## 3. Results

### 3.1. Aerosol Characterization

The aerosol was characterized with regard to temporal stability, mean particle size, number concentration, morphology, and chemical composition of the volatile organic compounds. With the measured physical quantities, the mass concentration can be calculated, assuming particle density and shape are known. 

The temporal stability of the dosage of each material is shown in Supplementary Figures S2 and S3 and proved to be very high. There are fluctuations and short breakdowns in some experiments, but these did not have a negative impact on the total test duration of 4 h, since 1 g/h of nanocomposite was dosed relatively consistently in each experiment, even though there were fluctuations over time.

Regarding chemical composition, Supplementary Figure S1A,B show SEM pictures of aerosols after TiO_2_ NP and PE + TiO_2_ NP incineration, indicating that nanocomposite incineration results in organic as well as inorganic matter. Unfortunately, additional chemical analyses of metal-based particles, such as ICP-MS or AAS, were not possible since the mass quantities on membranes after ALI exposure were below the detection limit of suitable analytical methods. However, a comparison of Supplementary Figure S1A,B indicates the appearance of pristine nanomaterial (Figure S1A) in the incineration-generated aerosol (Figure S1B). With regard to organic matter, particle-bound PAHs that are presumably being formed during thermal decomposition of the native PE matrix and collected on a glass fiber filter were submitted to solvent extraction and chemically characterized. Results showed the presence of six out of 16 priority PAHs, according to the US EPA (anthracene, fluoranthene, benz[*a*]anthracene, chrysene, benzo[*b*]fluoranthene, and dibenz(a,h)anthracene).

#### 3.1.1. Impact of Thermal Decomposition on the Aerosols Applied on A549 Cells

The nanoparticle suspensions were injected into the burner, where in the cases of TiO_2_ and CuO a stable suspension prepared in deionized water was applied. For preparation of CNT, gum arabic was used as a stabilizer.

**Figure 2:**
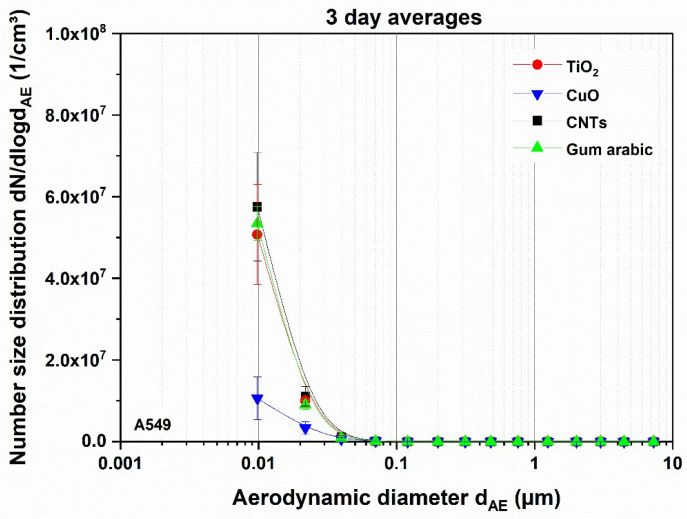
Average measured number size distributions of the different nanomaterial suspensions downstream of the thermal treatment, as determined via ELPI, subsequently applied to A549 cells. Error bars indicate standard deviation derived from three independently performed experiments.

In Figure 2, the averages of the measured number size distributions of the respective nanomaterials of three experiments are shown. Comparing the measured sizes downstream of the burner with the manufacturer’s specifications of the respective materials, particles were considerably smaller within this study. This effect has been described previously [36,37], but is still the subject of current investigations. The total number concentration was very high and the particle sizes so small that not the entire peak was within the measuring range of the ELPI. Since the CNTs are essentially carbon, it is expected that no CNTs would be visible downstream of the burner. This hypothesis was verified by the peak after thermal decomposition of pure gum arabic, which is the same as that seen after CNT combustion. Therefore, the stabilizer attributed to combustion products of the CNT suspension.

**Figure 3:**
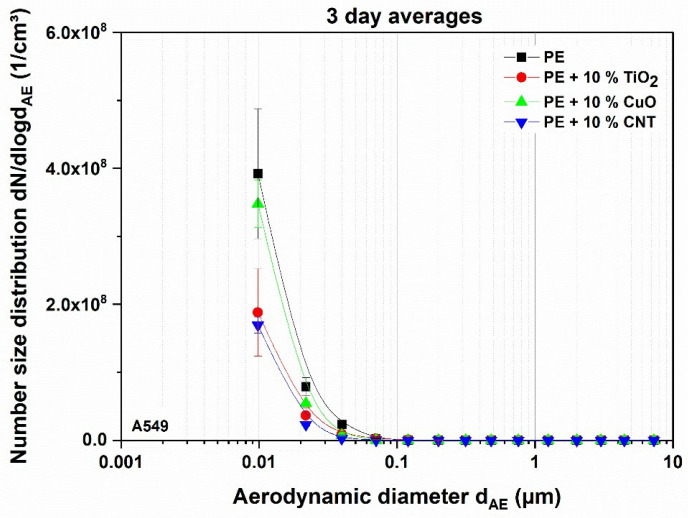
Average number size distribution of nanocomposite materials downstream of the thermal treatment, as determined via ELPI, subsequently applied to A549 cells. Error bars indicate standard deviation derived from three independently performed experiments.

The temporal stability of the combustion aerosols of all nanocomposite materials can be seen in Supplementary Figure S2, where the moving average of 300 data points (corresponding to a measurement time of 5 min) of the measured number concentration over time is shown. For example, the combustion of pure polyethylene led to a high number concentration, which was stable over time and different experiments. The material reservoir of the rotating brush generator was filled in the same way for each experiment, but since it has to be done manually there can be slight differences in the local packing density. Therefore, the dosage may end before the experiment is over, as can be seen on day 2 in Supplementary Figure S2. Nevertheless, the same mass of material is dosed over time; only the temporal distribution may vary. In Figure 3 the average number size distributions of the tested nanocomposites are shown.

#### 3.1.2. Impact of Thermal Decomposition on the Aerosols Applied on Reconstituted Tissue

**Figure 4:**
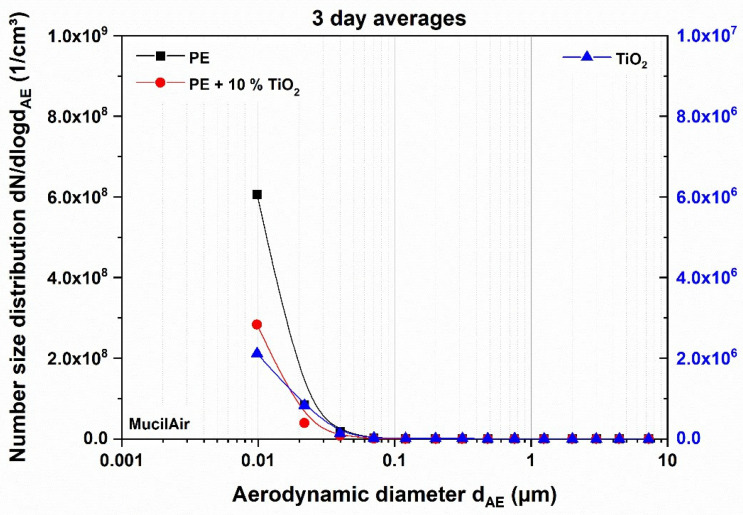
Average number size distributions of nanocomposite and nanoparticle materials downstream of the thermal treatment, tested in MucilAir™ tissue. The polymer-based materials refer to the left axis, the TiO_2_ particles to the right axis.

Figure 4 displays the average number size distributions of the materials applied on MucilAir™ tissue. The number concentration of PE and its composite are two orders of magnitude higher than the number concentration of the TiO_2_ particles. Therefore, the measurements cannot be displayed on the same linear scale and the nanocomposite in the diagram refers to the left ordinate and the nanoparticles to the right ordinate.

### 3.2. Dosimetry of Deposited Particles on Test Systems

The measured number concentration of the ELPI was converted into a mass concentration and an applied dose as calculated previously. The data are shown in Table 1. In general, the deposited dose was in a range of 18–280 ng/cm², but the small standard deviation shows that the experimental conditions were stable. The presence of NP in the PE matrix tends to reduce PM2.5 emissions compared to the pure matrix.

### 3.3. Characterization and Concentration of Combustion-Generated Volatile Organic Compounds

The components of the PE combustion were detected and quantified using TENAX TA sampling and subsequent TDS-GC-MS. They consisted mainly of volatile (VOC) and SVOC compounds. For the VOC, a concentration of 161 µg/m³, consisting mainly of alcohols (1-dodecanol), phenols, aldehydes (benzaldehyde), and unsaturated hydrocarbons were found. Besides higher-chained aliphatic compounds, SVOC revealed the appearance of 2,5-diphenylfuran and 2,5-diphenyl-1,4-benzoquinone. At 1580 µg/m³, the concentration of SVOC was almost 10 times higher than the concentrations of the VOC.

### 3.4. LDH Release by Combustion Aerosols

**Figure 5:**
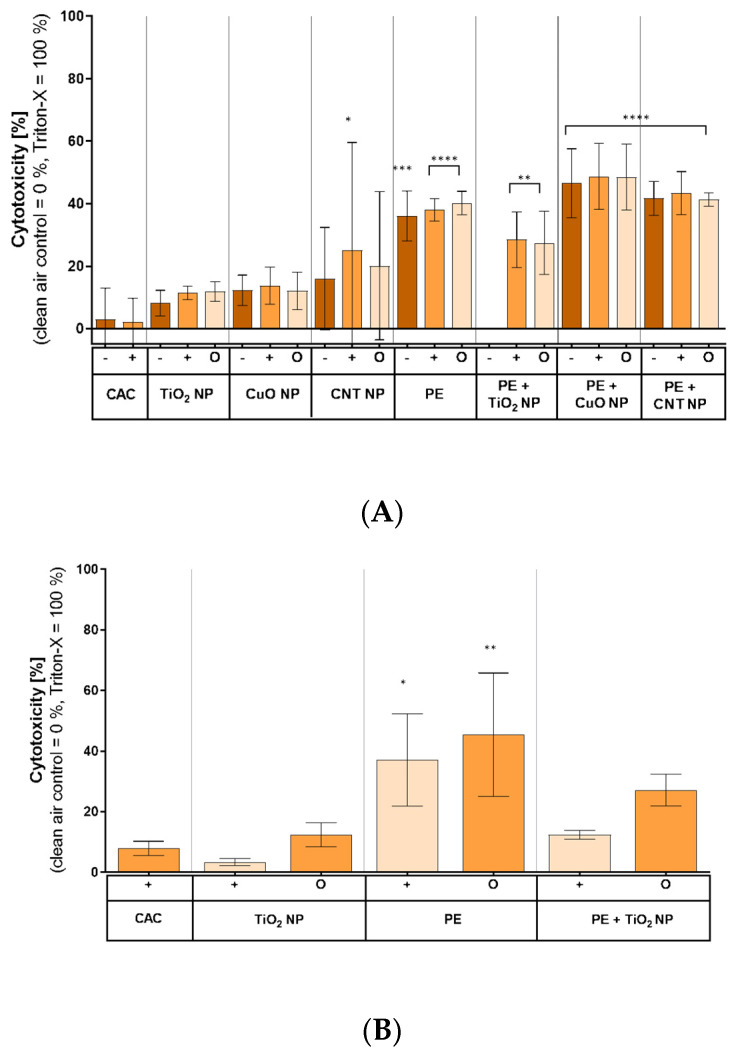
LDH release of A549 cells (**A**) and MucilAir™ tissue (**B**) after exposure to combustion-generated aerosols. Both cell systems were exposed to aerosols under conditions of normal particle deposition (−), increased particle deposition (+), and a filtered aerosol without particle fraction (O) for 4 h, as previously described, and incubated for 20 h. The 0.1% Triton X-100 incubated for 10 min was used as positive control for total cell lysis. Shown are the mean values of at least three independent experiments (*n* = 3) ± standard deviation (SD). Statistically significant by using one-way analysis of variance (ANOVA) followed by post hoc Dunnett’s test; * *p* ≤ 0.05; ** *p* ≤ 0.01; *** *p* ≤ 0.005; **** *p* ≤ 0.001. -: normal aerosol exposure; +: exposure under enhanced particle deposition; O: exposure of a filtered aerosol.

In order to investigate potential cytotoxic effects of aerosols, the membrane integrity was determined measuring LDH release in A549 cells and MucilAir™ test systems (Figure 5). In A549 cells exposed to pure, thermal-treated nanomaterials, no significant increase in LDH release was observed compared to clean air controls (CACs) (Figure 5A). In contrast, exposures toward aerosols after incineration of pure PE as well as of PE-based nanocomposites revealed a significant induction of cytotoxicity in A549 cells. The lowest LDH release (27%) was observed after exposure to PE + TiO_2_ NP, while the exposure to combustion aerosols of PE + CuO NP resulted in a higher LDH release (49%) compared to controls. None of the experiments revealed a difference between the exposures to an aerosol under conditions of normal particle deposition (−), increased particle deposition (+), and a filtered aerosol without particle fraction (O). Due to unexpected problems during the exposure campaign, it was not possible to obtain cytotoxicity data for the exposure to aerosols of PE + TiO_2_ NP incineration under conditions of normal particle deposition (−). However, it is suggested that the missing data would be similar to the other exposures since the exposure under enhanced particle deposition also did not show any effect. Regarding the MucilAir™ test system, compared to CAC there was no enhanced LDH release after the exposure to the combustion aerosol composed of pure TiO_2_ NP with or without filtration apparent (Figure 5B). The exposure toward aerosols after pure PE incineration resulted in an increased LDH release of up to 45%. However, these results exerted a high standard deviation between independent experiments. The exposure to aerosols after PE + TiO_2_ NP incineration showed no clear cytotoxic effects under conditions of enhanced particle deposition (+). In contrast, the exposure to the filtered aerosol of the same combustion material resulted in a distinct LDH release of 27%.

### 3.5. Transcriptional Toxicity Profile

**Figure 6:**
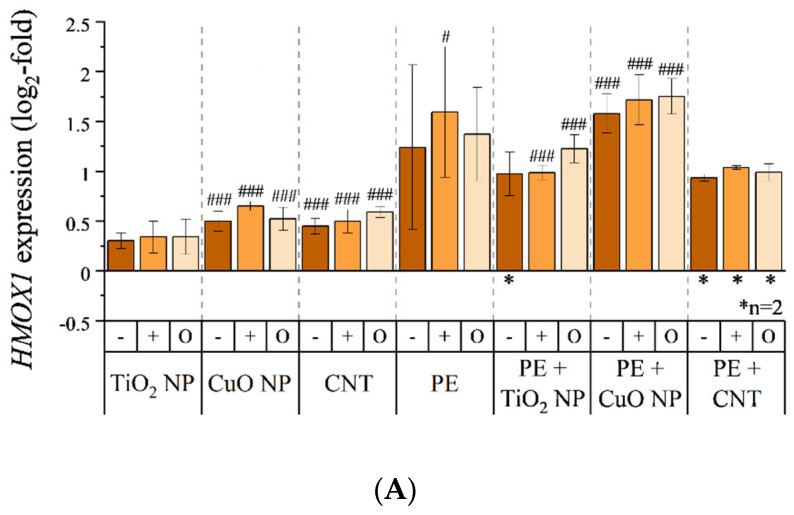

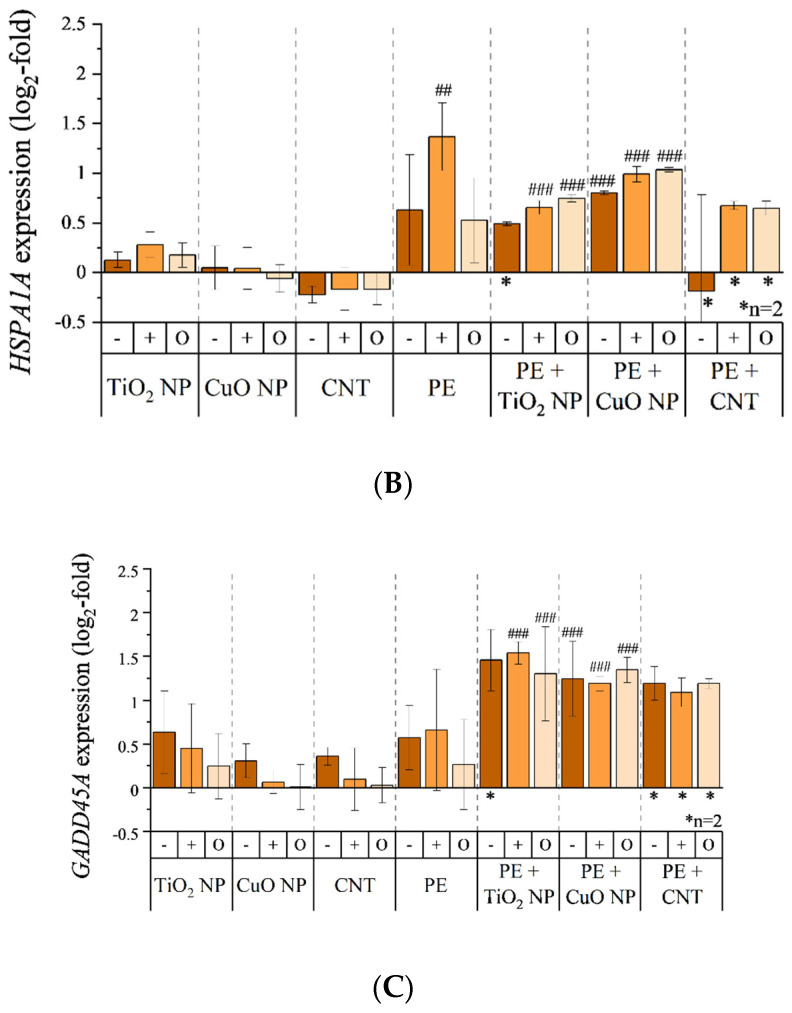
Impact of combustion-generated aerosols on *HMOX1* (**A**), *HSP1A1* (**B**), and *GADD45A* (**C**) expression in A549 cells. A549 cells were exposed to aerosols under conditions of normal particle deposition (−), increased particle deposition (+), and a filtered aerosol without particle fraction (O) for 4 h, as previously described, and incubated for another 20 h afterward. Thereafter, RNA was isolated and a high throughput RT-qPCR performed. Relative gene expression alterations are depicted as log_2_-fold change. If not stated otherwise (*), mean values of at least three independent experiments (*n* = 3) ± SD are shown. Statistically significant by using one-way analysis of variance (ANOVA), followed by post hoc Dunnett’s test: # *p* ≤ 0.05; ## *p* ≤ 0.01; ### *p* ≤ 0.001. -: normal aerosol exposure; +: exposure under enhanced particle deposition; O: exposure of a filtered aerosol.

An overview on the impact of all incineration aerosols on the transcriptional toxicity profile within both cell systems is depicted as a heatmap in Supplementary Figure S4. Briefly, only a few genes were altered in their expression after an aerosol exposure, provided that an expression alteration considered relevant is indicated by a ±1-log_2_ fold change (linear increased or decreased expression by factor 2). Regarding A549 cells, the most affected genes (*HMOX1*, *HSP1A1*, and *GADD45A*) are all associated with either oxidative or genotoxic stress (Figure 6). Furthermore, *DDIT3* showed an enhanced expression. However, it was not possible to obtain expression data of this gene in all experiments; therefore, this gene was not further considered within this study. While *HMOX1* expression was only slightly enhanced by aerosols of sole nanomaterial incineration (Figure 6A), expression was roughly doubled (+1-log_2_ fold change) after an exposure toward aerosols of PE or PE-containing nanocomposite incineration. Taking standard deviations into account, there was again no difference between the exposure scenarios of normal particle deposition (−), high particle deposition (+), and the sole gaseous fraction (O) within the respective materials. Also, the expression of *HSP1A1* showed no biological relevant alteration after exposure to aerosols of sole nanomaterial incineration (Figure 6B). In contrast to *HMOX1* expression, transcription of *HSP1A1* was less induced after exposure to PE-containing material incineration aerosols of PE. In addition, it was apparent that exposure to an aerosol after incineration of PE under conditions of high particle deposition (+) provoked a more pronounced gene expression compared to an exposure under conditions of normal particle deposition (−) or the sole gaseous phase (O). Considering standard deviations, all further exposures indicated no substantial difference between scenarios of normal particle deposition, high particle deposition, or applying the sole gaseous fraction within the respective materials. Expression of the genotoxic stress marker *GADD45A* was slightly increased after exposure to aerosols after sole nanomaterial incineration (Figure 6C). Exposure to aerosols of PE-containing material incineration once again resulted in an enhanced gene expression, even though incineration of PE itself resulted in a comparatively low *GADD45A* induction. The *GADD45A* expression was elevated relatively constantly to a 1.3-log_2_ fold change (2.5 x-fold change) after incineration of PE-containing nanocomposites. This was the only instance where a nano-scaled filler effect was observed within this study. Again, no relevant differences between total aerosol exposure (− and +) and exposure to the sole gaseous fraction of the aerosol (O) were observed within the respective materials. Regarding alterations in the gene expression profile of MucilAir™ tissue, only one gene (*IL8*) was affected (Supplementary Figure S4). Once again, the aerosol of sole TiO_2_ NP incineration showed no impact on gene expression while aerosols of PE or PE-containing nanocomposite incineration increased gene expression (Supplementary Figure S5). Again, when considering standard deviations, there was no difference between exposures to aerosols and the respective gaseous fractions after filtration.

### 3.6. Genotoxicity of Combustion Aerosols

**Figure 7:**
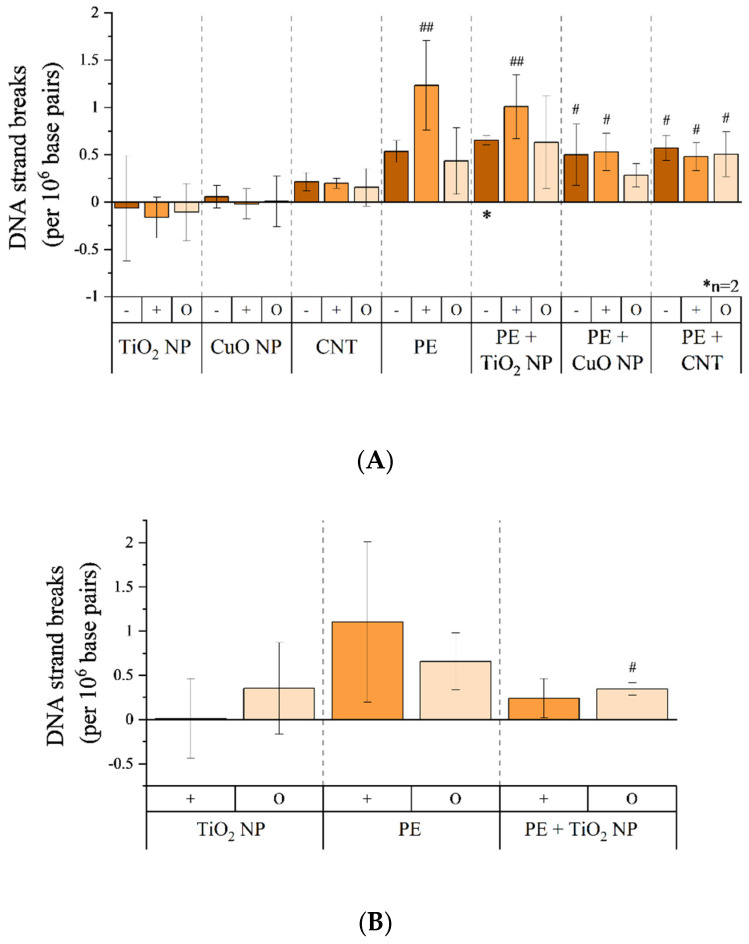
Induction of DNA strand breaks in A549 cells (**A**) and MucilAir™ (**B**) tissue by combustion-generated aerosols. Both cell systems were exposed to aerosols under conditions of normal particle deposition (−), increased particle deposition (+), and a filtered aerosol without particle fraction (O) for 4 h, as previously described, and incubated for another 20 h afterward. Subsequently, alkaline unwinding was performed to quantify DNA strand breaks. Results were normalized to a CAC without high voltage (normal particle deposition conditions) for A549 cells and on CAC with high voltage (high particle deposition conditions) for MucilAir™ tissues. Menadione (100 µM) incubated for 1 h was used as a positive control to induce DNA strand breaks (Supplementary Figure S6). If not stated otherwise (*), the mean values of at least three independent experiments (*n* = 3) ± standard deviation (SD) are shown. Statistically significant by using one-way analysis of variance (ANOVA), followed by post hoc Dunnett’s test; # *p* ≤ 0.05; ## *p* ≤ 0.01. −: normal aerosol exposure; +: exposure under enhanced particle deposition; O: exposure of a filtered aerosol.

Potential genotoxic effects of combustion generated aerosols were analyzed using alkaline unwinding and thus quantifying DNA strand breaks (Figure 7). The exposure to incineration-generated aerosol of sole nanomaterials did not induce any substantial increase in DNA strand breaks in A549 cells (Figure 7A). However, also at this endpoint, exposure toward aerosols of PE or PE-containing nanocomposite incineration resulted in an increased genotoxic effect. The exposure to these aerosols induced roughly 0.5 DNA strand breaks per 10^6^ bp, independent of the nano-scaled filler. However, conditions of high particle deposition resulted in enhanced DNA strand break formation after exposure toward incineration aerosols of PE itself and PE + TiO_2_ NP. Genotoxic effects were also apparent in MucilAir™ tissues (Figure 7B). Again, the exposure to an aerosol of sole TiO_2_ NP incineration did not indicate any adverse effects. Regarding the filtered aerosol of TiO_2_ NP incineration, a slight enhanced amount of DNA strand breaks was observed, which was considered as not relevant taking standard deviations into account. Exposure to a PE incineration aerosol resulted in the most pronounced effect of 1.1 DNA strand breaks per 10^6^ bp. Using an upstream filter, the amount of DNA strand breaks was slightly reduced, however, these data exerted some variations. The aerosol of PE + TiO_2_ NP incineration induced no substantial amounts of DNA strand breaks compared to CAC. The induction of strand breaks induced by high voltage, the CNT suspension stabilizer, and positive controls are depicted in Supplementary Figure S6.

### 3.7. Inflammatory Response on Combustion-Generated Aerosols

**Figure 8:**
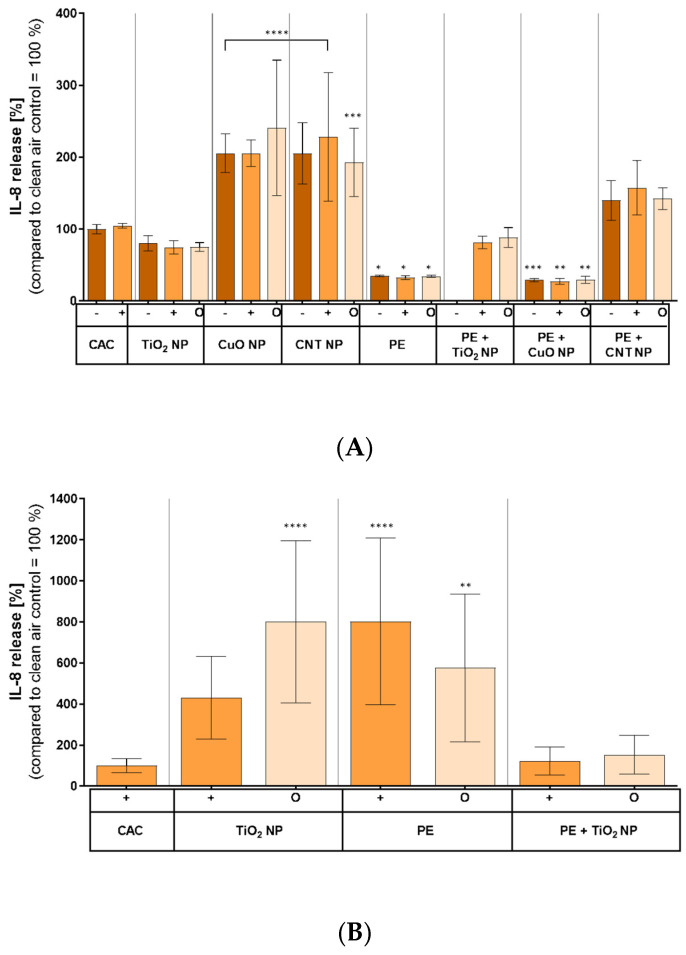
Inflammatory response of A549 cells (**A**) and MucilAir™ tissue (**B**) 24 h after exposure to combustion-generated aerosols compared to a clean air control (100%). Both cell systems were exposed to the correspondent aerosols for 4 h, as previously described, and incubated for another 20 h afterward, following IL-8 detection in basal media. Clean air controls exerted IL-8 levels of about 2000 pg/mL on average. Shown are the mean values of at least three independent experiments (*n* = 3) ± standard deviation (SD). Statistically significant by using one-way analysis of variance (ANOVA), followed by post hoc Dunnett’s test; * *p* ≤ 0.05; ** *p* ≤ 0.01; *** *p* ≤ 0.005; **** *p* ≤ 0.001. −: normal aerosol exposure; +: exposure under enhanced particle deposition; O: exposure of a filtered aerosol.

The inflammatory response of A549 cells (Figure 8A) and MucilAir™ tissue (Figure 8B) to combustion aerosols was assessed by measuring IL-8 release as a widely used indicator (Figure 8). In A549 cells, an increase in IL-8 release after exposure to combustion aerosols of CuO NP and CNT compared to the control was observed (Figure 8A). The exposure toward aerosols after TiO_2_ NP incineration resulted in a decreased IL-8 release of about 20% compared to the control. Similarly, the exposure of A549 cells to combustion aerosols of pure PE, PE + TiO_2_ NP, and PE + CuO NP led to a decrease in IL-8 release of up to 40% compared to the control. In contrast, an inflammatory response in A549 cells was observed after exposure to aerosols of PE + CNT NP incineration. None of the experiments showed differences between the different exposure conditions.

When exposing MucilAir™ to combustion aerosols of TiO_2_ NP or PE, both treatments led to an increase in IL-8 release compared to the control; however, no effect was seen for the combustion aerosols of TiO_2_ NP-enabled PE.

## 4. Discussion

To the best of the authors’ knowledge, this study was the first to investigate the toxicological effects of well characterized aerosols released during combustion of thermoplastic nanocomposites using an air–liquid interface exposure system. Even though studies on the toxicological potential of combustion-generated particulate matter [1,7,12,13] as well as VOCs [10,11,12] have been published, none of them was designed to investigate the effect of the native aerosol using appropriate realistic lung cell culture models. In the current study we investigated the combustion behavior of PE-based nanocomposites on a lab-scale burner. As nano-scaled fillers TiO_2_ NP, CuO NP, as well as CNT were chosen for this study, with TiO_2_ NP representing a commonly used insoluble and inert nanomaterial, CuO NP as a known in vitro cyto- as well as genotoxic nanomaterial, and CNT as a fiber-shaped nanomaterial.

The burner used in this study was operated with a stoichiometric ethylene-air flame, resulting in very high temperatures of approximately 2000 °C. Such high temperatures are not found in waste incineration plants, which represent the real end-of-life scenario of nano-enabled thermoplastics. The temperature in a typical waste incineration plant can reach up to 1100 °C (grate furnace) [14], whereas the temperature in a hazardous waste incineration plant can reach up to 1400 °C (rotary kiln furnace). However, with this lab-scale setup it was possible to carry out reproducible and well-controlled experiments, which primarily aimed to establish the respective methods combined with the ALI exposure system to obtain toxicological data, and which could now be used with other test rigs up to large-scale plants. 

Within the present study, dose determination was carried out by converting the measured number size distribution using an ELPI into a mass concentration. Since the measuring range of the ELPI ends at 10 µm, which corresponds to the PM_10_ inlet of the exposure station, potentially present larger particles do not have to be excluded from the size distribution. Nevertheless, this method can be prone to errors because, for example, the density of the particles must be known. However, the effective density of nanoparticles and their agglomerates can differ considerably from the bulk density. For example it has been shown that internal voids have an impact on the effective density, which resulted in a wide range (513 up to 804 kg/m³) for TiO_2_ NP [38], indicating the variation between densities of NP and bulk material as well as the need for proper material characterization. Since the TEM images of the experiments with the nano-scaled fillers showed that the aerosols consist of approximately spherical, individual particles, the respective bulk density could be used for these exposures. In the case of nanocomposites, it was much more difficult since the material was strongly changed by combustion and not only the polymer, but also various degradation products from the polymer were present. For this reason, the density 1 g/cm³ was used in all experiments with nanocomposites. This may not indicate the exposure dose correctly, but using this approach resulted in comparable calculations.

A further difficulty in determining the exposure dose is the assumption of a deposition efficiency resulting from the diffusion of the particles. This deposition efficiency was determined experimentally [39], but for another aerosol, and therefore the result cannot necessarily be transferred to all investigated aerosols. The increase in deposition rate due to the application of an electric field was also determined experimentally [19,40]. However, the aerosol used for this purpose contained significantly larger particles than those used in this study. In general, larger particles can carry more charges, which allows the dose to be significantly increased when an electric field is applied. As a consequence, as there was a significant amount of nano-scaled particles that can generally carry fewer charges, the increase in deposition efficiency may have been overestimated here.

Regarding the potential adverse effects of aerosols formed during combustion of the pure nano-scaled fillers, no genotoxicity or transcriptional changes but cytotoxicity and inflammatory response for CuO NT and CNT were observed in A549 cells. In previous studies, toxicity of the applied TiO_2_ NP species P25 (Evonik/Degussa) has been investigated after submerged incubation as well as upon air–liquid interface exposure. Briefly, cyto- and genotoxic effects of TiO_2_ NP in A549 cells were only observed at high concentrations and incubation periods over 24 h under submerged conditions [41,42,43,44]. Data after ALI exposure have been contradictory. On the one hand, a comparable toxicity pattern to submerged exposure has been reported applying relatively low doses of 3 µg/cm² not indicating any cytotoxicity [45] and higher doses of 25 µg/cm² impairing metabolic activity [46,47]. On the other hand, neither cell viability nor genomic stability or gene expression were affected by TiO_2_ NP in one study, even at high doses [29], while low doses induced cyto- and genotoxic effects as well as pro-inflammatory transcriptional changes in another study [27]. So far, the impact of TiO_2_ NP on MucilAir™ tissue has not been published, but other metal oxides such as CeO_2_ and CuO were applied using an ALI exposure system [48,49,50]. Regarding largely insoluble CeO_2_ NP, which might be comparable to TiO_2_ NP in their mode of action, no cytotoxicity, DNA damage, or relevant gene expression changes were apparent while oxidative stress was slightly increased [49]. In our study, under the applied TiO_2_ NP dose of 73 ng/cm² in A549 cells and 10.9 µg/cm² for MucilAir™ tissues, no cytotoxic effects were observed. However, MucilAir™ tissues showed an inflammatory response to the TiO_2_ NP by releasing cytokine IL-8. Cyto- and genotoxicity of CuO NP in A549 cells were reported via submerged conditions in a number of studies [28,51,52,53] and also after ALI exposure [29,47,54,55]. In addition, changes on a transcriptional level were investigated using the same gene expression profiling approach under submerged and ALI exposure conditions [28,29]. Except for Jing et al., all other studies using ALI exposure observed adverse effect after applying concentrations of 5 µg/cm² or higher, which explains the lack of adverse effects within the present study. Comparing the applied doses to the literature, the dose within this study under conditions of enhanced particle deposition (136 ng/cm²) is comparable to 150 ng/cm², which resulted in an enhanced LDH release by Jing et al. [55]. However, using a normalization to the negative control and not, as commonly applied, a normalization to a positive control, data analysis of LDH release was processed differently by Jing and colleagues. Evaluating our data in the same way also shows an enhanced LDH leakage that is, however, not biologically relevant. In contrast, combustion of CuO NP induced an inflammatory response (IL-8) in A549 cells. Interestingly, incineration of CuO NP as nano-scaled filler in PE thermoplastic reduced this inflammatory response.

Regarding the more physiological MucilAir™ tissue system, the impact of CuO NP after ALI exposure was studied comprehensively by Kooter and colleagues [48,50]. In concordance with these studies, we observed minor cytotoxic effects of less than 25% LDH release. An inflammatory response as well as an impact on gene expression was observed in both published studies in a dose-dependent manner. Furthermore, Kooter and colleagues identified potential biomarkers for metal/metal oxide nanoparticles using RNA microarrays [48]. However, the applied doses of CuO NP within these studies were higher (lowest dose of 1.2 µg/cm²) than in the present study (136 ng/cm² at high particle deposition), which might explain the lack of transcriptional response after CuO NP exposure within this study.

In contrast to inorganic particles, CNT and CNT fragments were not suspected to be found after incineration at high temperatures, as already observed in different studies [1,56]. This observation supports the lack of adverse effects in A549 cells by CNTs in our study regarding cytotoxicity. However, combusted CNTs induced an inflammatory response in A549 cells, even as a nano-scaled filler in PE thermoplastic. This inflammatory response of lung cells may be explained by the presence of certain trace metals like aluminum, iron, or molybdenum found in CNTs. Even if CNTs were investigated as aerosols, no cytotoxicity was seen at high doses after ALI exposure of MucilAir™ tissue [57]. In our study, the increase in LDH release after the exposure to aerosols of PE and PE-based nanocomposite combustion indicated that toxic gaseous substances, e.g., CO, VOC, and SVOC, are deliberated during the incineration process, which was also proven analytically by TDS-GC-MS analysis, revealing organic compounds VOC and SVOC within this study. To distinguish between toxic effects due to released particles or generated toxic gases, upstream filters were installed to remove any particles from the aerosol. Since no difference between exposure to aerosols and the gaseous phase was apparent, it was concluded that the gaseous phase was solely responsible for all the toxic effects observed. Taking the applied exposure doses into account, the particle deposition, even by HV, might have been not high enough to induce any particle-driven toxicity. The induction of toxic gases, including VOCs and SVOCs, was not only shown within this study, but also previously in various publications, e.g., [10,11,12]. These generated toxic gases, VOCs, and SVOCs can, according to the literature, re-condensate to secondary particles, which might represent the majority of particles during incineration of PE and its nanocomposites [9]. On the other side, VOCs or SVOCs can react directly with human lung cells as volatile compounds. This was shown in a previous investigation using toluene and benzene as model VOCs under ALI exposure conditions. Both compounds induced cytotoxicity, inflammation, oxidative stress, and genotoxicity as volatiles [58]. These effects are supported by the observation in the present study showing for most of the combustion experiments with thermoplastics or nano-enabled thermoplastics enhanced cytotoxicity and induced expression of oxidative (*HMOX1, HSPA1A*) and genotoxic (*GADD45A*) stress marker genes, inflammatory response, and the induction of DNA strand breaks. The latter might be induced by cellular uptake of combustion-generated PAHs, which have been detected, possibly bound to formed secondary particles. A similar observation was made by Dilger and colleagues, who observed PAHs adsorbed to wood smoke particles [59]. This might also explain the increase of DNA strand breaks in A549 cells after particle enhancement during pure PE and PE + TiO_2_ NP incineration. For these exposures the calculated deposited dose was at least increased by a factor of 2 compared to all other A549 exposures experiments.

In conclusion, the obtained results support the existing knowledge on the release of nano-scaled fillers after incineration of nano-enabled thermoplastics. Furthermore, it is suggested that the observed adverse effects in human lung cells or tissues were almost exclusively caused by organic compounds like toxic gases, VOCs, and SVOCs or their secondary organic aerosols, which are generated during incomplete combustion of the nano-particle carriers.

## Figures and Tables

**Table 1:** Mass concentrations and applied doses for pure diffusional deposition (dose) and increased electrostatic deposition (dose HV, high voltage). Given are averages and standard deviation of three independently conducted experiments.

Material	Mass Concentration (µg/cm³)	Dose (ng/cm²)	Dose HV (ng/cm²)	Cell System
TiO_2_	137 ± 20	15 ± 2	73 ± 11	A549
CuO	256 ± 151	27 ± 16	136 ± 80	A549
CNT (+ Gum arabic)	44 ± 7	5 ± 1	23 ± 4	A549
Gum arabic	34 ± 4	4 ± 0,4	18 ± 2	A549
PE	505 ± 8	54 ± 1	268 ± 4	A549
PE + TiO_2_	527 ± 317	56 ± 34	280 ± 168	A549
PE + CuO	235 ± 18	25 ± 2	125 ± 10	A549
PE + CNTs	106 ± 16	11 ± 2	54 ± 8	A549
PE	465 ± 120	48 ± 12	238 ± 58	MucilAir™
PE + TiO_2_	209 ± 27	22 ± 3	111 ± 14	MucilAir™
TiO_2_	20,610 ± 801	2186 ± 361	10,932 ± 1803	MucilAir™

## Data Availability

The data presented in this study are available on request from the first (MH, NM) and corresponding authors (AH, DS) for researchers of academic institutes who meet the criteria for access to the confidential data.

## Supplementary Materials

The following are available online at https://www.mdpi.com/article/10.3390/nano11071685/s1, Figure S1. SEM and TEM pictures of pure and processed particles. A: SEM picture of TiO_2_ particles and B: soot of PE + TiO_2_ NP nanocomposite incineration that was deposited on the impaction stages of the ELPI for subsequent analysis. C: TEM pictures of different particles that were deposited on the impaction stages of the ELPI for subsequent analysis. Supplementary Figure S2. Number concentration of the dosed nanoparticles (left side) and nanocomposites (right side) downstream of the burner measured via ELPI. Supplementary Figure S3. Number concentration of the dosed nanoparticles and nanocomposites downstream of the burner measured via ELPI. Supplementary Figure S4. Overview of the gene expression profile of A549 cells and MucilAir™ tissue after exposure to combustion-generated aerosols. Supplementary Figure S5. IL8 expression in MucilAir™ tissue after an exposure to combustion-generated aerosols. Supplementary Figure S6. Background of DNA strand breaks in A549 cells (a) and MucilAir™ tissue (b) using exposure to clean air and a stabilizer for CNT stock solution.
