# Supplementary material for: Impact of Nanocomposite Combustion Aerosols on A549 Cells and a 3D Airway Model
Source: Nanomaterials (Basel). 2021 Jun 27;11(7):1685. doi: 10.3390/nano11071685 (PMC8304990; doi:10.3390/nano11071685)
Supplement: Supplementary file 1 [file nanomaterials-11-01685-s001.zip › nanomaterials-1261090-SI.pdf]

Supplementary Materials

Impact of Nanocomposite Combustion Aerosols on A549 Cells and a 3D Airway Model

Matthias Hufnagel [1,†], Nadine May [2,†], Johanna Wall [1], Nadja Wingert [3], Manuel Garcia-Käufer [3], Ali Arif [3], Christof Hübner [4], Markus Berger [5], Sonja Mülhopt [2], Werner Baumann [2], Frederik Weis [6], Tobias Krebs [5], Wolfgang Becker [4], Richard Gminski [3], Dieter Stapf [2,*] and Andrea Hartwig [1,*]

[1] Department of Food Chemistry and Toxicology, Institute of Applied Biosciences, Karlsruhe Institute of Technology, 76131 Karlsruhe, Germany; Matthias.Hufnagel@gmail.com (M.H.); Johanna.Wall@kit.edu (J.W.)

[2] Institute for Technical Chemistry, Karlsruhe Institute of Technology, 76344 Eggenstein-Leopoldshafen, Germany; nadine.may@kit.edu (N.M.); sonja.muelhopt@kit.edu (S.M.); werner.baumann@kit.edu (W.B.)

[3] Institute for Infection Prevention and Hospital Epidemiology, Medical Center—University of Freiburg, Faculty of Medicine, University of Freiburg, 79110 Freiburg, Germany; nadja.wingert@uniklinik-freiburg.de (N.W.); manuel.garcia-kaeufer@uniklinik-freiburg.de (M.G.-K.); ali.arif@uniklinik-freiburg.de (A.A.); richard.gminski@uniklinik-freiburg.de (R.G.)

[4] Fraunhofer Institute of Chemical Technology, 76327 Pfinztal, Germany; christof.huebner@ict.fraunhofer.de (C.H.); wolfgang.becker@ict.fraunhofer.de (W.B.)

[5] Vitrocell® Systems GmbH, 79183 Waldkirch, Germany; m.berger@vitrocell.com (M.B.); t.krebs@vitrocell.com (T.K.)

[6] Palas GmbH, 76229 Karlsruhe, Germany; frederik.weis@palas.de

* Correspondence: dieter.stapf@kit.edu (D.S.); andrea.hartwig@kit.edu (A.H.)

† These authors contributed equally to this work.

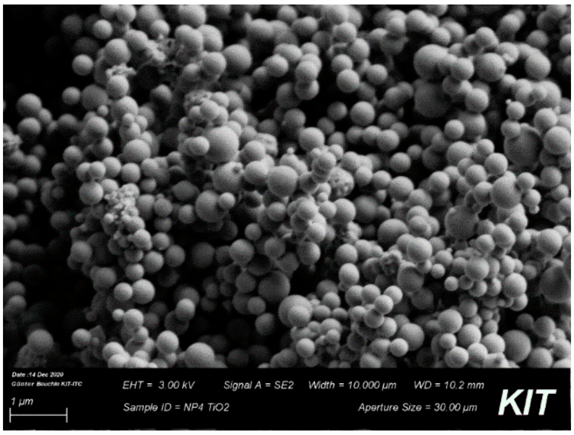

(a)

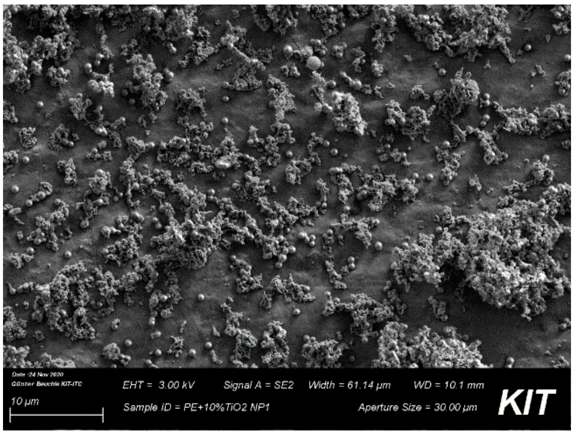

(b)

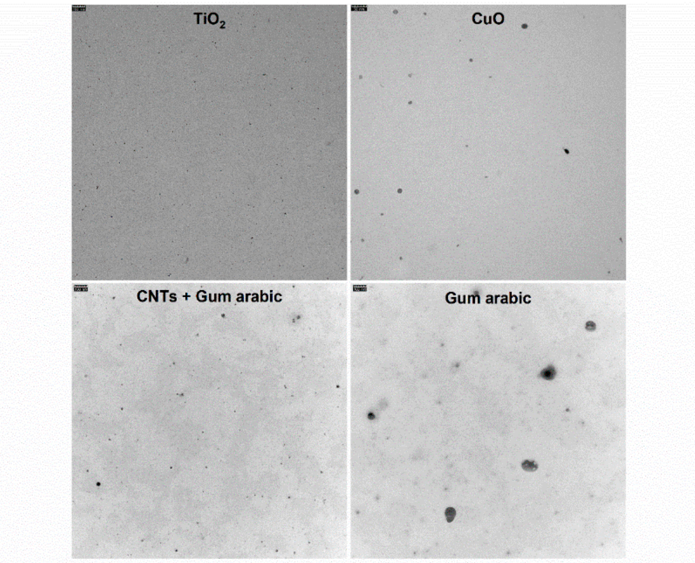

(c)

Supplementary Figure S1. SEM and TEM pictures of pure and processed particles. (**a**) SEM picture of pure TiO_2 aerosol particles that were deposited on the impaction stages of the ELPI for subsequent analysis. (**b**) SEM picture of particles from combustion of PE + 10% TiO_2 that were deposited on the impaction stages of the ELPI for subsequent analysis. (**c**) TEM pictures of different particles that were deposited on the impaction stages of the ELPI for subsequent analysis.

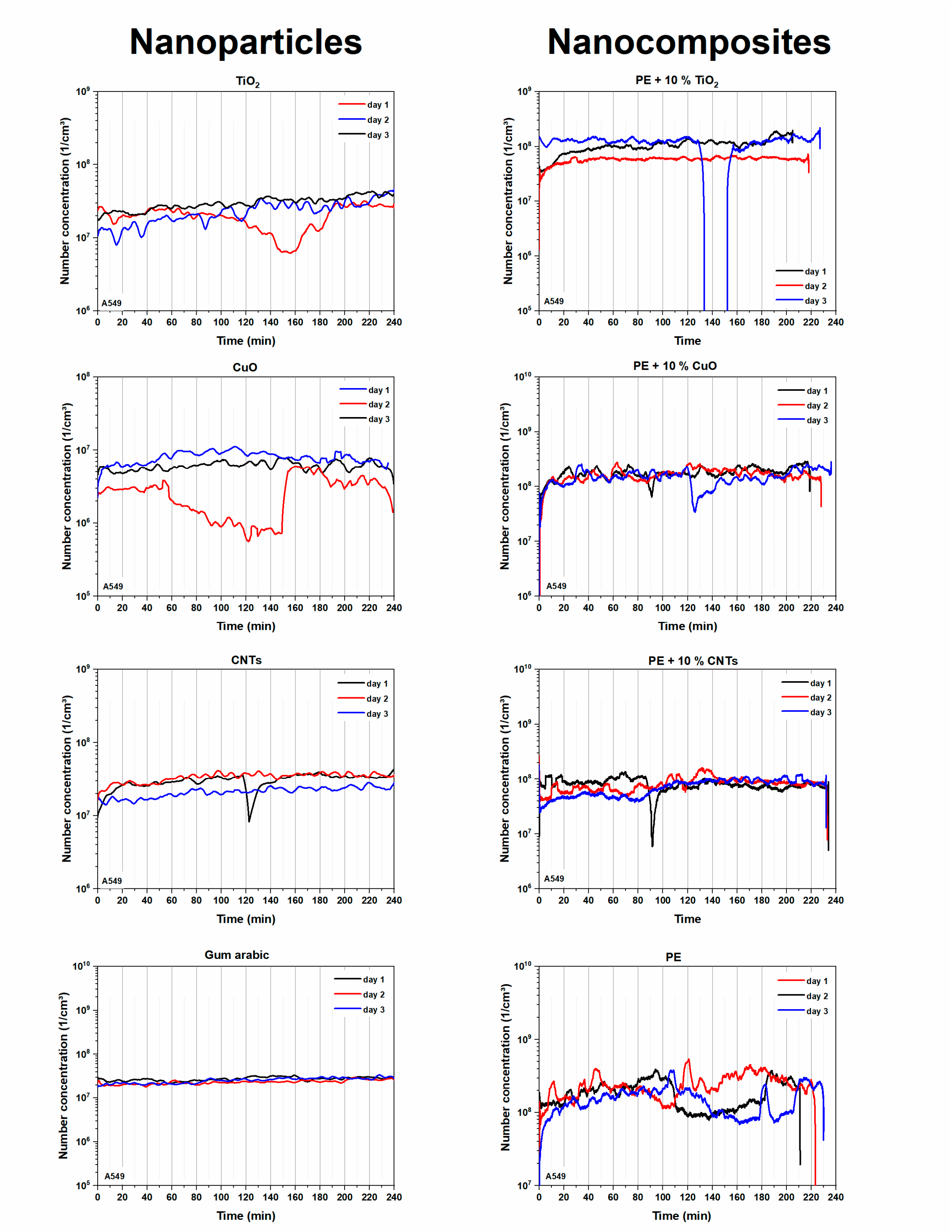

Supplementary Figure S2. Number concentrations of the dosed nanoparticles (left side) and nanocomposites (right side) downstream of the burner measured via ELPI. The three different colors indicate three different experiments with A549 cells, each of which with a duration of exposure of 4 h.

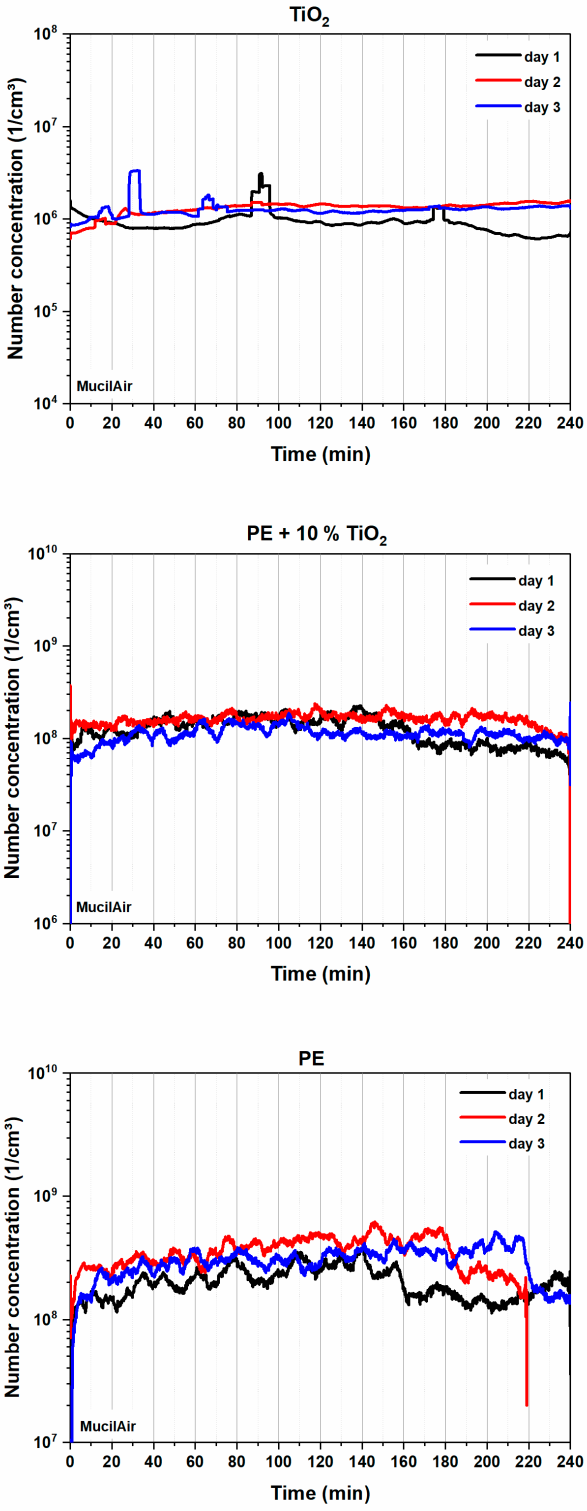

Supplementary Figure S3. Number concentrations of the dosed nanoparticles and nanocomposites downstream of the burner measured via ELPI. The three different colors indicate three different experiments with MucilAir™ tissue, each of which with a duration of exposure of 4 h.

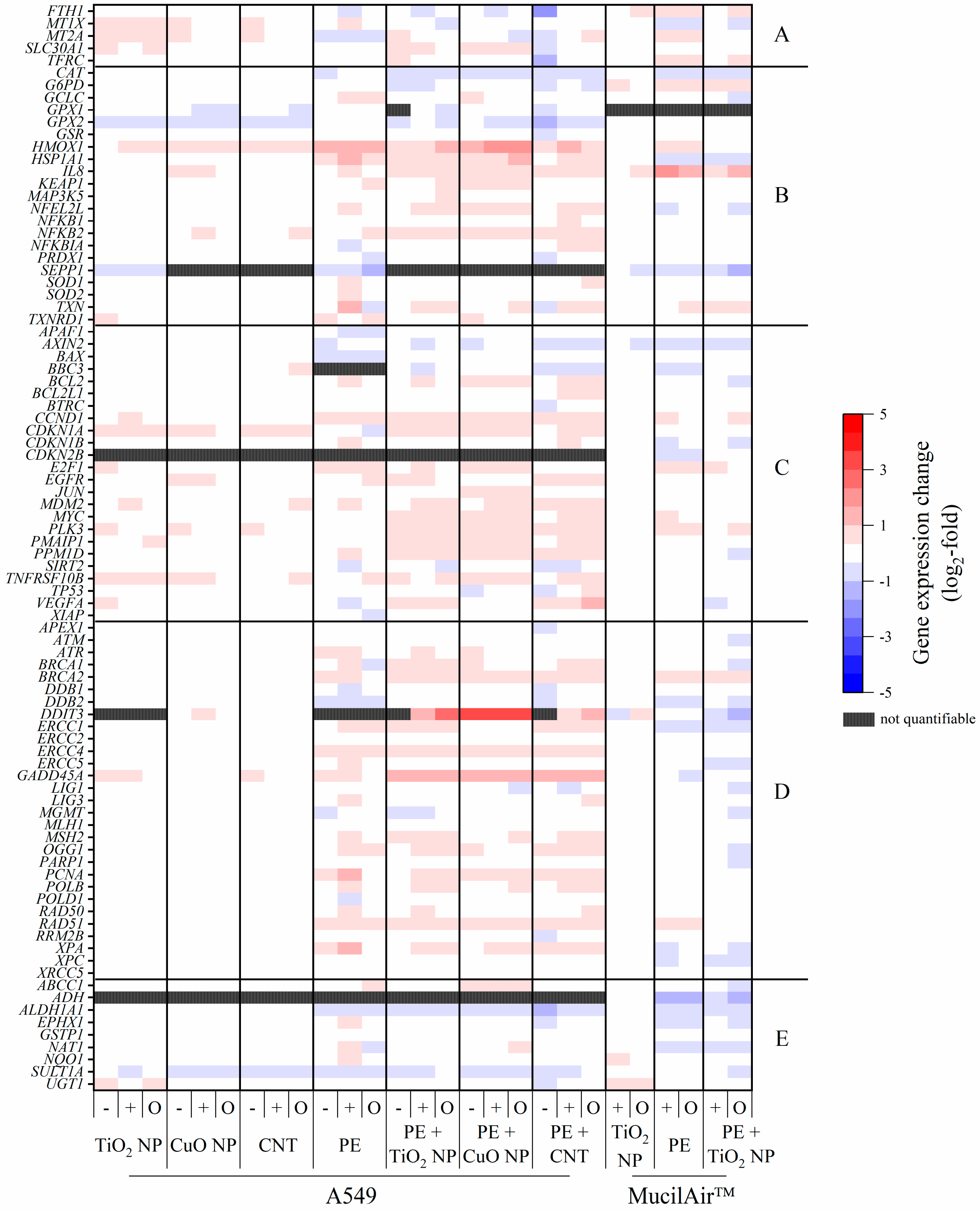

Supplementary Figure S4. Overview on the gene expression profile of A549 cells and MucilAir™ tissue after exposure to combustion generated aerosols. Both cell systems were exposed to the respective aerosols for 4 h as previously described and incubated for another 20 h afterwards. Thereafter, RNA was isolated and a high throughput RT-qPCR performed. Relative gene expression alterations are depicted as log_2-fold change, with red indicating an enhanced gene expression and blue indicating attenuated gene expression. Displayed is the expression of genes associated with metal homeostasis (A), oxidative stress response (B), apoptosis and cell cycle regulation (C), DNA damage response and repair (D) as well as xenobiotic metabolism (E). If not stated otherwise, mean values of at least three independent experiments (n=3) ± SD are shown. −: normal aerosol exposure, +: exposure under enhanced particle deposition, O: exposure of a filtered aerosol.

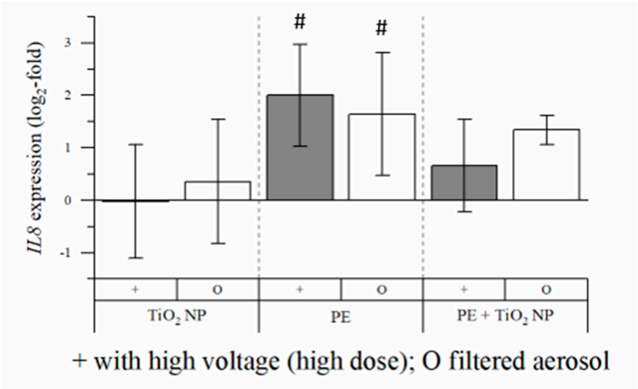

Supplementary Figure S5. *IL8* expression in MucilAir™ tissue after an exposure to combustion generated aerosols. MucilAir™ tissues were exposed to the respective aerosols for 4 h as previously described and incubated for another 20 h afterwards. Thereafter, RNA was isolated and a high throughput RT-qPCR performed. Relative gene expression alterations are depicted as log_2-fold change. If not stated otherwise, mean values of at least three independent experiments (n=3) ± SD are shown. Statistically significant by using one-way analysis of variance (ANOVA), followed by post hoc Dunnett's test: # $p \leq 0.05$; ## $p \leq 0.01$; ### $p \leq 0.001$. +: exposure under enhanced particle deposition; O: exposure of a filtered aerosol.

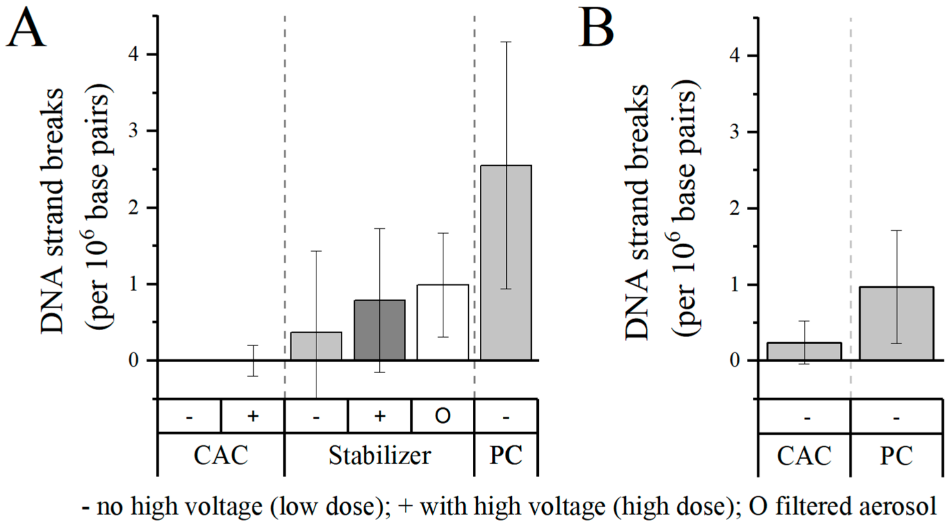

Supplementary Figure S6. Background of DNA strand breaks in A549 cells (**A**) and MucilAir™ tissue (**B**) using exposure to clean air and a stabilizer for CNT stock solution. Both cell systems were exposed to clean or an aerosol of stabilizer (Gummi arabicum) combustion for 4 h as previously described and incubated for another 20 h afterwards. Subsequently, Alkaline Unwinding was performed to quantify DNA strand breaks. Results were normalized to an CAC without high voltage (normal particle deposition conditions) for A549 cells and on CAC with high voltage (high particle deposition conditions) for MucilAir™ tissues. Shown are at least the mean values of three independent experiments (n=3) ± standard deviation (SD). -: normal aerosol exposure, +: exposure under enhanced particle deposition, O: exposure of a filtered aerosol
